# Rif2 Promotes a Telomere Fold-Back Structure through Rpd3L Recruitment in Budding Yeast

**DOI:** 10.1371/journal.pgen.1002960

**Published:** 2012-09-20

**Authors:** Heiko Poschke, Martina Dees, Michael Chang, Sandeep Amberkar, Lars Kaderali, Rodney Rothstein, Brian Luke

**Affiliations:** 1 Zentrum für Molekulare Biologie der Universität Heidelberg, DKFZ-ZMBH Allianz, Heidelberg, Germany; 2 Department of Genetics and Development, Columbia University Medical Center, New York, New York, United States of America; 3 Technische Universität Dresden, Medical Faculty, Institute for Medical Informatics and Biometry, Dresden, Germany; The University of North Carolina at Chapel Hill, United States of America

## Abstract

Using a genome-wide screening approach, we have established the genetic requirements for proper telomere structure in *Saccharomyces cerevisiae*. We uncovered 112 genes, many of which have not previously been implicated in telomere function, that are required to form a fold-back structure at chromosome ends. Among other biological processes, lysine deacetylation, through the Rpd3L, Rpd3S, and Hda1 complexes, emerged as being a critical regulator of telomere structure. The telomeric-bound protein, Rif2, was also found to promote a telomere fold-back through the recruitment of Rpd3L to telomeres. In the absence of Rpd3 function, telomeres have an increased susceptibility to nucleolytic degradation, telomere loss, and the initiation of premature senescence, suggesting that an Rpd3-mediated structure may have protective functions. Together these data reveal that multiple genetic pathways may directly or indirectly impinge on telomere structure, thus broadening the potential targets available to manipulate telomere function.

## Author Summary

Impaired telomere elongation eventually results in telomere dysfunction and can lead to diseases such as dyskeratosis congenita, which is associated with bone-marrow failure and pulmonary fibrosis. Cancer cells require continuous telomere maintenance to ensure continued cellular proliferation. Therefore the regulation of telomere function, both positively (in the case of dyskeratosis congenita) and negatively (for cancer), may be of therapeutic benefit. In this study we have used yeast to determine which genetic factors are important for a certain telomeric structure (the loop structure), which may help to maintain chromosome ends in a protected state. We found that multiple genetic factors and pathways affect telomere structure, ranging from metabolic signaling to specific telomere-binding proteins. We found that proper chromatin structure at the telomere is essential to maintain a telomere fold-back structure. Importantly, there was a strong correlation between telomere structure and function, as the mutants found in our screen (looping defective) were often associated with rapid senescence and telomere dysfunction phenotypes. We believe that, through the regulation of the various genetic pathways uncovered in our screen, one may be able to both positively and negatively influence telomere function.

## Introduction

The physical ends of linear chromosomes resemble double-strand breaks (DSBs) in many respects with the exception that DSBs result in the activation of the DNA damage response and are eventually subject to repair; activities to which telomeres are refractory [1]. This essential quality of telomeres exists as a result of their repetitive sequence that is bound by specific proteins (shelterin and CST complexes), which in turn inhibit DNA damage checkpoints, DNA repair activities and exonuclease-mediated degradation [2]–[3]. In yeast, the CST (Cdc13-Stn1-Ten1) complex is essential for viability and prevents the accessibility of 5′ exonucleases (primarily Exo1) to the telomere [4]–[6]. Upon inactivation of CST with temperature-sensitive alleles of *CDC13* and *STN1*, cells undergo a DNA damage-mediated checkpoint arrest due to the accumulation of single-stranded (ss) telomeric DNA [6]–[8]. In parallel, the Rap1, Rif1 and Rif2 complex, also contribute to telomere end protection by limiting telomeric ssDNA accumulation and subsequent checkpoint activation [9]–[10].

In most human somatic cells, telomeres shorten during each cell division due, in part, to the end-replication problem [11]–[12]. Eventually, the loss of telomeric DNA leads to telomere dysfunction, checkpoint activation and cellular senescence. Some cell types as well as most cancer cells avoid telomere attrition-induced senescence by expressing the specialized reverse transcriptase, telomerase. Telomerase elongates telomeres through the iterative addition of short sequence repeats to the 3′ ends of telomeres, compensating for the end-replication problem [12]. Wild type *S. cerevisiae* constitutively express telomerase, however the cellular senescence phenotype can be induced following its inactivation/deletion [13].

In yeast, reporter genes become silenced when placed in the vicinity of telomeres [14]. This telomere-induced silencing is dependent on the Sir2/3/4 lysine deacetylation (KDAC) complex, which is recruited to chromosome ends via the telomere binding protein, Rap1 [15]. Apart from the Sir2/3/4 complex, other KDACs also contribute to the heterochromatic constitution of telomeres and sub-telomeres. The class I KDAC, Rpd3 (the yeast ortholog of human KDAC1), consisting of two sub-complexes, Rpd3L and Rpd3S [16], also localizes to telomeres and is important to establish the euchromatin/heterochromatin boundary in the sub-telomeric regions [17], as well as to prevent hyper-silencing [18]. The class II KDAC, Hda1, also contributes to chromatin regulation at yeast telomeres [19]. The relationships between heterochromatin and telomere structure/function remain unclear.

It has been postulated that telomere protection may stem, in part, from a higher-order chromatin structure. Analysis of telomeric DNA from human and mouse cells has revealed that the telomere terminus can be hidden in a lariat-like structure termed a t-loop [2], [20]–[21]. T-loops, thought to form through the strand invasion of the 3′ telomeric overhang into the double-stranded region of the telomere, have also been found in chickens, worms, plants, and protozoa [22]–[25]. Via electron microscopy, telomeric loops have also been observed in yeast (*K. lactis*) with over-elongated telomeres [26], and the telomere associated *S. pombe* protein, Taz1, has been shown to re-model model DNA substrates into t-loops [27]. However, due to the small size of yeast telomeres it has been difficult to both prepare and analyze wild type length yeast telomeres via electron microscopy [26]. In the budding yeast, *S. cerevisiae*, both genetic and chromatin immunoprecipitation-based experiments have revealed that wild type telomeres do fold-back onto themselves and into the subtelomeric region [26], [28]–[30], suggesting that loops or fold-back structures are indeed important for telomere function in yeast. Apart from the Sir2/3/4 deacetylase complex in budding yeast being important for this fold-back [29], and the shelterin component, TRF2 in human cells being required for t-loop formation [20], [25], the regulation of such telomeric structures remains poorly understood. In this study we have taken an unbiased genome-wide screening approach in yeast to better understand how telomere structure/fold-back is regulated *in vivo*. We demonstrate that multiple biological processes influence telomere structure, including the state of the subtelomeric heterochromatin as dictated by multiple lysine deacetylases. Furthermore, we find that there are direct correlations between the inability of a telomere to fold-back and telomere dysfunction, implying that the loop structure may make important contributions to telomere protection.

## Results

### A genetic screen reveals mutants required for telomere fold-back

**Figure 1:**
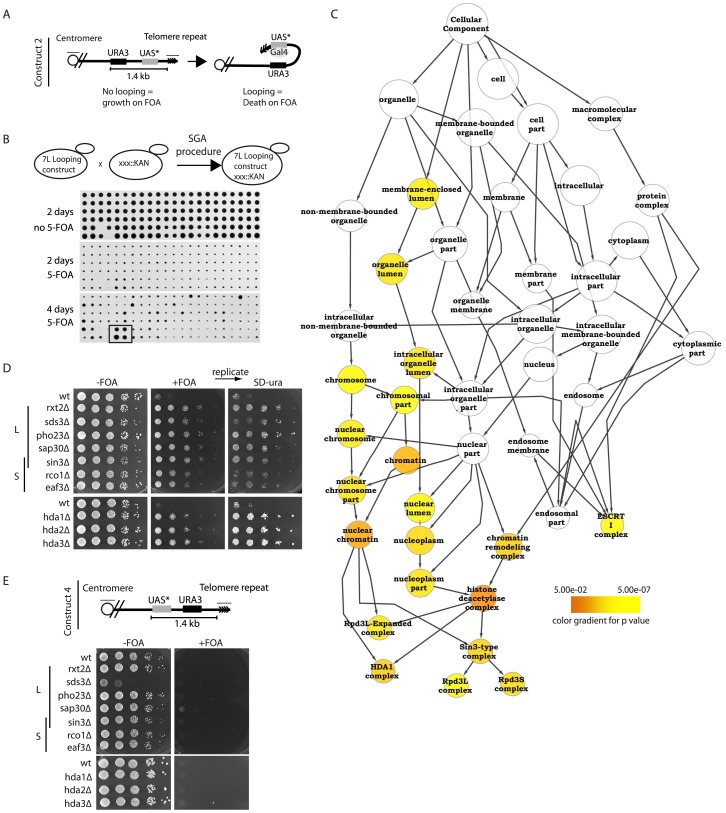
Histone deacetylation is required for proper telomere structure. (A) Construct 2 consists of the *URA3* gene followed by a downstream Gal UAS with a mutated TATA box (UAS*). When integrated at telomere 7L, the UAS* folds back and drives *URA3* transcription in the presence of galactose. (B) Using the SGA (synthetic genetic array) technology, construct 2 was introduced into the ∼4800 strains of the viable haploid yeast gene deletion collection. Subsequently cells were robotically pinned onto −FOA and +FOA galactose-containing media in quadruplicate and scored for growth. A positively scoring hit is highlighted (box in bottom panel) as an example of a non-looping mutant. (C) Validated hits were analyzed using the cytoscape plugin, BinGO, which created a tree of significantly enriched GO (gene ontology) processes over background. (D) All indicated deletion mutants within the Rpd3L, Rpd3S and Hda1 complexes were re-constructed and spotted onto the indicated media in 10-fold serial dilutions following an overnight culture in YPD to confirm the looping defects identified in the high-throughput screen. Plates were incubated 2–3 days before being imaged. +FOA plates were subsequently replica plated onto SD-URA media to ensure that construct was not lost or mutated. (E) For construct 4, the UAS* was placed in front of the *URA3* gene and subsequently integrated at telomere 7L (top). Cell spottings were performed exactly as described in (D) with the indicated genotypes.

By placing a TATA-less galactose-inducible UAS (upstream activating sequence) downstream of the *URA3* gene (from hereon referred to as construct 2), *URA3* transcription is only achieved when the UAS loops back and comes into proximity with the *URA3* promoter [28]–[29] (Figure 1A). Fold-back-induced transcription only takes place when this construct is integrated at the telomere and does not occur when it is integrated at an internal chromosomal locus [29]. Transcription of *URA3* results in lethality on media containing the drug 5-fluoroortic acid (5-FOA), providing a robust readout (cell death on 5-FOA) for successful telomere looping.

To better understand how the telomere fold-back structure in yeast is regulated, we introduced construct 2 into the yeast haploid deletion collection using the synthetic genetic array (SGA) procedure [31], resulting in the construction of ∼4800 haploid deletion mutants harboring construct 2 (Figure 1B). Robotic pinning of these strains in quadruplicate onto galactose media in the presence and absence of 5-FOA revealed potential looping defective mutants that grew on 5-FOA (Figure 1B, bottom panel example of looping defective mutant). All positively scoring mutants were independently re-constructed and spotted as serial dilutions onto galactose +/− 5-FOA media in duplicate. We confirmed 112 yeast mutants that were defective for telomere looping and subsequently ranked them qualitatively for growth on 5-FOA (Table 1, Figure S1A). Using the Cytoscape BinGO plugin [32], the statistically over-represented GO (gene ontology) categories were determined for our positive scoring candidates (Figure 1C). The confirmed mutants formed the “positive hit set” whereas the “reference set” consisted of all 4800 genes screened. The analysis used a hypergeometric test and significance was tested at 5% (*p*<0.05) after applying Benjamini & Hochberg False Discovery Rate (FDR) correction for multiple testing. This protocol revealed histone deacetylation as a significantly enriched GO term in the GO-Cellular Component and the GO-Biological Process ontologies. Moreover, the Rpd3L, Rpd3S and Hda1 KDAC complexes were specifically over-represented (Figure 1C, Table 1). We introduced the looping construct into deletion mutants of all members of the Rpd3L/S and Hda1 complexes (including those that did not score positive in the screen) and determined that all complex members tested were important for wild type-like telomere structure (Figure 1D). Importantly, we replicated the 5-FOA plates onto media lacking uracil to ensure that the FOA resistance observed was not due to inactivation of the *URA3* gene (Figure 1D). FOA resistant strains maintain the ability to grow on media lacking uracil due to low basal levels of the *URA3* transcript (see Figure S1D). To confirm that there was not an inherent problem of inducing transcription within the subtelomere of these mutants, we generated and introduced construct 4 (Figure 1E) into Rpd3L, Rpd3S and Hda1 mutants where the TATA-less UAS was placed upstream of *URA3* and found that (unlike with construct 2) upon galactose induction all mutants were dead on 5-FOA containing media (Figure 1E). Figure 1E demonstrates that in the mutants of the Rpd3L/S and Hda1 complexes, if the UAS in construct 2 were able to loop back to the *URA3* promoter it would be able to induce transcription to an extent that would result in cell death on FOA, as is the case with wild type cells. We excluded that telomere length variation may affect the looping read-out in the *rpd3Δ* and *hda1Δ* mutants, as no significant changes in telomere length were detectable when comparing the KDAC mutants to isogenic wild type cells (Figure S1B, S1C). Finally, we demonstrated that the plate read-out effects that we have observed with construct 2 on 5-FOA are due to changes in levels of the *URA3* transcript (Figure S1D) as has previously been reported [29] and not an unrelated artifact of 5-FOA.

**Table 1:** Mutants from the screen that grew in FOA indicating a telomere looping defect.

Gene	ORF	Defect	Overlap	Gene	ORF	Defect	Overlap	Gene	ORF	Defect	Overlap
URM1	YIL008W	+++		**MRT4**	**YKL009W**	**+++**	**C**	RTG1	YOL067C	++	
TIP41	YPR040W	+++		AMD1	YML035C	+++		**HDA2**	**YDR295C**	**++**	**C**
RPL27A	YHR010W	+++		**ASF1**	**YJL115W**	**+++**	**B**	**OCA5**	**YHL029C**	**++**	**A**
TKL1	YPR074C	+++		RIM21	YNL294C	+++		OCA6	YDR067C	++	
**HDA1**	**YNL021W**	**+++**	**B**	**RIF1**	**YBR275C**	**+++**	**C,D,F**	**LEO1**	**YOR123C**	**++**	**D,E**
**TRK1**	**YJL129C**	**+++**	**C**	SLM5	YCR024C	+++		ALO1	YML086C	+	
**SAP30**	**YMR263W**	**+++**	**C**	**HTD2**	**YHR067W**	**+++**	**E**	PET130	YJL023C	+	
SET2	YJL168C	+++		LYS14	YDR034C	+++		**HIT1**	**YJR055W**	**+**	**D**
**RPB9**	**YGL070C**	**+++**	**C**	Unknown	YJR119C	+++		MRPL9	YGR220C	+	
BUD31	YCR063W	+++		NPC2	YDL046W	+++		VTC3	YPL019C	+	
**CTK1**	**YKL139W**	**+++**	**C**	RTG3	YBL103C	+++		MSR1	YHR091C	+	
**SIN3**	**YOL004W**	**+++**	**B,C**	TPM2	YIL138C	+++		**EDE1**	**YBL047C**	**+**	**E**
EAF3	YPR023C	+++		**SUR4**	**YLR372W**	**+++**	**C**	KRE28	YDR532C	+	
BER1	YLR412W	+++		KGD1	YIL125W	+++		MAL13	YGR288W	+	
PPM1	YDR435C	+++		**NUT1**	**YGL151W**	**+++**	**C**	**PCP1**	**YGR101W**	**+**	**C**
**SEC28**	**YIL076W**	**+++**	**B**	**SWR1**	**YDR334W**	**+++**	**A**	**ELP4**	**YPL101W**	**+**	**A,B**
**VPS28**	**YPL065W**	**+++**	**C,D**	UBP1	YDL122W	+++		BUD25	YER014C-A	+	
**MED1**	**YPR070W**	**+++**	**B**	GCN2	YDR283C	+++		UBR1	YGR184C	+	
GUF1	YLR289W	++		SSF1	YHR066W	++		SWI4	YER111C	+	
unknown	YDL073W	++		RPL24B	YGR148C	++		ACO2	YJL200C	+	
**RRD1**	**YIL153W**	**++**	**A,E**	**SPE1**	**YKL184W**	**++**	**A**	**PDX3**	**YBR035C**	**+**	**C**
GCR2	YNL199C	++		LSM1	YJL124C	++		IDH1	YNL037C	+	
PUB1	YNL016W	++		RAV1	YJR033C	++		PUF6	YDR496C	+	
FKH2	YNL068C	++		NAP1	YKR048C	++		FMP49	YER038W-A	+	
TAL1	YLR354C	++		**ARP6**	**YLR085C**	**++**	**A,B**	**RIM101**	**YHL027W**	**+**	**E**
UBP3	YER151C	++		SUR2	YDR297W	++		GEP4	YHR100C	+	
TPS2	YDR074W	++		RSA3	YLR221C	++		**SKI3**	**YPR189W**	**+**	**E**
PIH1	YHR034C	++		MEP1	YGR121C	++		MSN4	YKL062W	+	
MKS1	YNL076W	++		**SPE3**	**YPR069C**	**++**	**A**	MHR1	YDR296W	+	
**YME1**	**YPR024W**	**++**	**E**	PTC6	YCR079W	++		unknown	YIL055C	+	
MSB3	YNL293W	++		**OCA1**	**YNL099C**	**++**	**A,E**	COS10	YNR075W	+	
RCO1	YMR075W	++		**SIW14**	**YNL032W**	**++**	**D**	IGO2	YHR132W-A	+	
PIN2	YOR104W	++		**OCA2**	**YNL056W**	**++**	**A**	CHS6	YJL099W	+	
STP2	YHR006W	++		MSC1	YML128C	++		**PEP8**	**YJL053W**	**+**	**A**
RPS24A	YER074W	++		SIF2	YBR103W	++		MVB12	YGR206W	+	
LDB16	YCL005W	++		INP53	YOR109W	++		ALO1	YML086C	+	
SRP40	YKR092C	++		TIR3	YIL011W	++		CBS2	YDR197W	+	
HDA3	YPR179C	++		PHO23	YNL097C	++					

(+++ = strong defect, ++ = medium defect, + = weak defect). Letter codes under “overlap” refers to publications where commonolaties with other genome wide telomere function screens were found (A = Addinall et al. [39], B = Chang et al. [41], C = Askree et al. [43], D = Gatbonton et al. [44], E = Addinall et al. [38], F = Xue et al. [49]).

In conclusion, an unbiased genome-wide screen has implicated lysine deacetylation through the Rpd3L, Rpd3S and Hda1 complexes in promoting a structural change (likely a fold-back) at budding yeast telomeres.

### Chromatin immunoprecipitation confirms telomere structural defects

**Figure 2:**
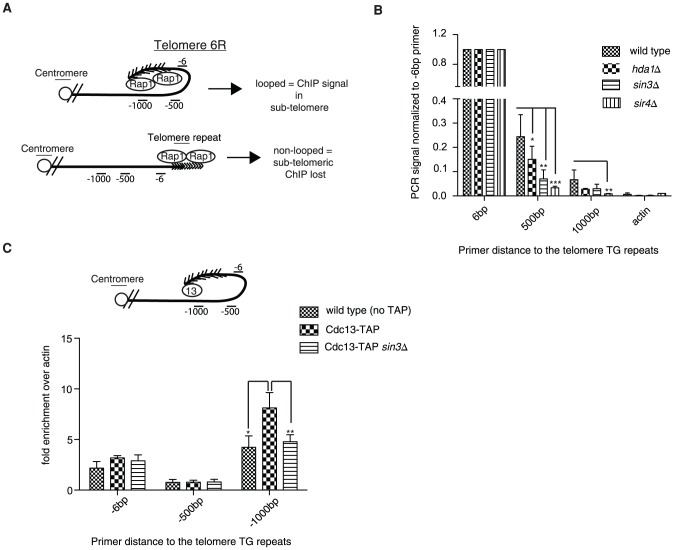
Chromatin immunoprecipitation confirms structural defects. (A) The immunoprecipitation of Rap1 following cross-linking should be associated with subtelomeric sequences at natural telomere 6R if the fold-back structure is intact (upper diagram); however the subtelomeric ChIP will be lost upon loop opening (lower diagram). (B) Upon Rap1 ChIP from exponentially growing cells, a subtelomeric signal was detected up to 1 kb away from the base of the telomeric repeats in wild type cells, whereas the signal was largely diminished in *hda1Δ*, *sin3Δ* and *sir4Δ* mutants. DNA stemming from the actin locus (*ACT1*) was not detected following Rap1 ChIP and was used as a background control. Error bars represent SD from three independent experiments. (C) Cdc13-TAP (13) was also able to precipitate subtelomeric DNA up to 1 kb away from the start of the telomeric sequence at telomere 6R following cross-linking (n = 3, error as SD) in comparison to wild type (non-tagged controls). This ChIP signal at -1000 was reduced to that of non-tagged controls in the *sin3Δ* strain. The difference in ChIP signal distribution between Rap1 (B) and Cdc13-TAP (C) is likely due to the different positioning of the two proteins on the telomere (compare diagrams in A and C for explanation). For all experiments above error bars represent SD of the mean from at least 3 independent experiments and * indicates statistically significant differences as determined through unpaired student's t-tests whereby * = p<0.05, ** = p<0.01, *** = p<0.001.

To demonstrate that the looping defect we observe is not specific to modified telomere 7L (construct 2), we employed a previously established chromatin immunoprecipitation (ChIP) technique where it has been shown at a natural telomere (telomere 6R) that α-Rap1 antibodies are able to precipitate subtelomeric DNA greater than 2 kb away from the start of the telomeric tract, despite the fact that chromatin is sheared into fragments of 0.5 kb [30]. From this study, it was concluded that the subtelomeric ChIP signal from cross-linked Rap1 extracts was a result of the telomere looping back into the subtelomeric region (Figure 2A, top). We predicted that the subtelomeric signal would be lost in mutants identified in our above-described screen (Figure 2A, bottom). In agreement with previous reports, we could detect cross-linked Rap1 at a position 0.5 kb and to a lesser extent 1 kb away from the subtelomere/telomere transition point in wild type cells, indicative of a telomeric loop-back structure (Figure 2B). Unlike the previous report [30], we did not detect reproducible differences at positions farther than 1.5 kb from the telomere (not shown), which is likely due to the smaller chromatin fragment size used in our ChIP protocol. Strikingly, the Rap1 signal was diminished in the subtelomere in *hda1Δ* mutants and lost to a greater extent in *sin3Δ* cells (Rpd3L/S common subunit), consistent with the 5-FOA assay using construct 2 (Figure 2B, Figure 1D). *sir4Δ* cells were used as a looping defective positive control for the Rap1 ChIP assay (Figure 2B) [29] and indeed displayed the greatest loss of Rap1 signal in the subtelomeric region. Importantly, the bulk of our chromatin was sheared to 0.3 kbp fragments or less, excluding the possibility that our subtelomeric signals come from inefficient sonication (Figure S2A). Furthermore, Rap1 protein levels were not affected in any of the above-mentioned mutant backgrounds (Figure S2B).

In order to rule out the unlikely possibility that Rap1 spreading into the subtelomere may account for a portion of the ChIP signal in the assay described above (Figure 2A), we repeated the ChIP experiments using an epitope-tagged Cdc13-TAP (Tandem Affinity Purification) allele. Cdc13 associates with the 3′ ssDNA telomeric overhang, and therefore is not prone to spread into the subtelomere. Furthermore we reasoned that by using Cdc13-TAP we would be able to more easily reconcile differences at the -1 kb position due to its distal positioning at the 3′end. Consistently we were also able to detect Cdc13-TAP associated with subtelomeric DNA 1000 bp upstream of the telomeric tract (indicative of a fold-back), and the signal was reduced to background levels in the *sin3Δ* mutant (Figure 2C). The *sin3Δ* mutation did not result in decreased Cdc13-TAP protein levels, which could have potentially accounted for the reduced ChIP signal (Figure S2C). It is important to note that we did not enrich significant amounts of subtelomeric DNA at the -6 and -500 bp positions following the Cdc13-TAP ChIP (Figure 2C) although Cdc13 has been previously shown by similar methods to localize to subtelomeres [33]. We interpret this to indicate that our sonication was extremely efficient and fragments above 400 bp (the approximate length of the wild type telomere) were extremely rare and did not give a signal significantly above background (untagged control). In order verify this notion we deleted telomerase (*EST2*) in Cdc13-TAP cells and let telomeres shorten over 25 and 50 generations (Figure S2D and S2E). We predicted that upon telomere shortening we would be able to increasingly detect a signal at the -6 position as telomeres would be shorter than our sheared chromatin fragments (see Figure 2C for visualization). Indeed, we found that as telomere length decreased to under 300 bp (our average chromatin fragment size) we were able to detect a robust Cdc13-TAP ChIP signal at the -6 position at natural telomere 6R (Figure S2F).

In summary we have confirmed that the looping defect observed in *hda1Δ* and *sin3Δ* (Rpd3L/S subunit) mutants using construct 2 and 5-FOA as a read-out (Figure 1D) can be recapitulated using an independent method (ChIP) at natural telomere 6R (Figure 2B, 2C).

### Rif2 promotes proper telomere structure through Rpd3L recruitment

**Figure 3:**
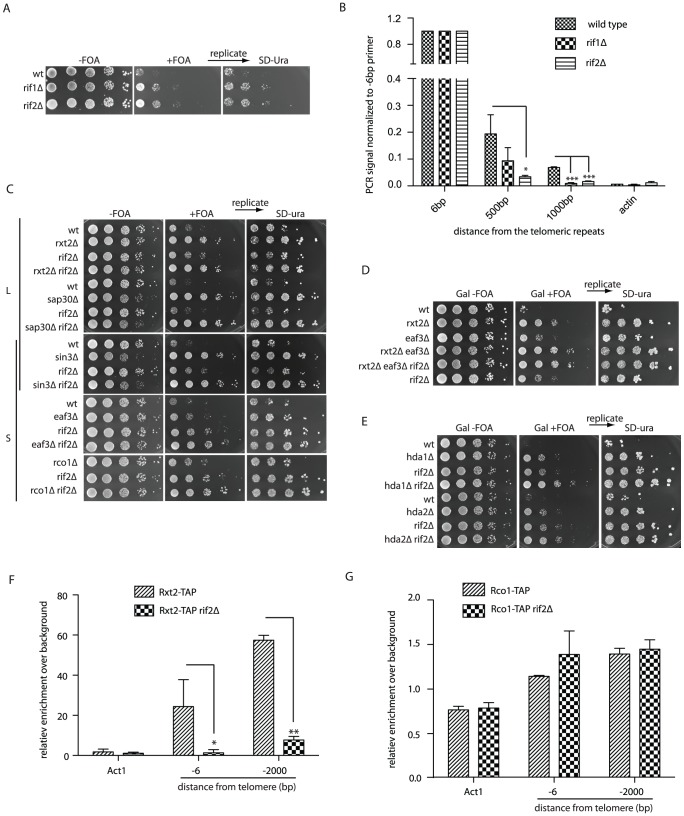
The Rif proteins promote Rpd3L recruitment to telomeres. (A) Cells with the indicated genotypes and harboring construct 2 were spotted onto the galactose media (+/− FOA) (B) Rap1 ChIP was performed as described for Figure 2B. The defect in looping of the *rif1Δ* and *rif2Δ* strains is reflected in the loss of Rap1 association to subtelomeric DNA following cross-linking (n = 3, error as SD). (C) Looping defects of the indicated mutants were assayed as in Figure 1D in order to assess genetic interactions between *rif2Δ* and the Rpd3L and Rpd3S complexes. All colonies were replicated from +FOA onto SD-URA plates to ensure that construct 2 was intact. (D) The looping defect in Rpd3L (*rxt2Δ*) mutants is additive when combined with Rpd3S mutations (*eaf3Δ*), and not further exacerbated by deletion of *RIF2*. (E) Both *hda1Δ rif2Δ* and *hda2Δ rif2Δ* double mutants have more severe looping defects than either of the single mutants as seen by their increased resistance to 5-FOA. (F and G) Cells expressing a TAP (tandem affinity purification) tagged version of either Rxt2-TAP (Rpd3L) or Rco1-TAP (Rpd3S) were cross-linked and DNA was precipitated with IgG beads. -6 bp and -2000 bp refer to the position of the reverse primer with respect to the beginning of the telomeric tract on telomere 6R, amplicons being on average approximately 100 bp. The Rpd3L complex is lost at telomeres in a *rif2Δ* mutant (F) whereas Rpd3S association is not affected (G). For all experiments above error bars represent SD of the mean from at least 3 independent experiments and * indicates statistically significant differences as determined through unpaired student's t-tests whereby * = p<0.05, ** = p<0.01, *** = p<0.001.

Among the list of looping defective mutants we were intrigued that *RIF1*, a regulator of telomere length, was also implicated in promoting a fold-back structure (Table 1). Since Rif2 works in parallel with Rif1 to regulate telomere length we introduced construct 2 into *rif2Δ* cells and found that like *rif1Δ* cells, *rif2Δ* mutants also displayed a looping defect (Figure 3A). Using the Rap1 ChIP assay (Figure 2A) it was also evident that both *rif1Δ* and *rif2Δ* mutants had structural defects in terms of folding back into the subtelomere (Figure 3B). As with the above described Rap1 ChIP, we confirmed that Rap1 protein levels were not altered in *rif1Δ* and *rif2Δ* cells which may have accounted for observed differences (Figure S3A). From here on we have performed further analysis only with the *rif2Δ* mutant rather that *rif1Δ* cells due to the fact that apart from telomere length regulation, Rif1 also plays an important role in telomere capping [10], checkpoint regulation [34]–[35] as well as telomere localization [36], which greatly complicated the interpretation of *rif1Δ* cells and their genetic interactions. Ongoing studies are directed at better understand the contributions of Rif1 in promoting the telomere fold-back structure.

We constructed double mutants between *rif2Δ* and mutants of the Rpd3L, Rpd3S and Hda1 complexes harboring construct 2 in order to assess potential genetic interactions between different pathways involved in telomere looping. Whereas the Rpd3S specific mutants (*eaf3Δ* and *rco1Δ*) displayed a slight additive growth advantage on 5-FOA when combined with *rif2Δ* mutants compared to the respective single mutants (Figure 3C, bottom panels) there were no additive effects with *rif2Δ* mutants and the Rpd3L complex (*sap30Δ* and *rxt2Δ*) (Figure 3C, top panel). *sin3Δ rif2Δ* double mutants were not additive in comparison to the respective single mutants (Figure 3C, middle panel), as Sin3 belongs to both L and S complexes. As expected, *rxt2Δ* (Rpd3L) and *eaf3Δ* (Rpd3S) double mutants had a slight additive looping defect in comparison to the single mutants and further deletion of *RIF2* did not exacerbate this defect (Figure 3D). The looping defect of *hda1Δ* and *hda2Δ* mutants was also additive with *rif2Δ* mutants (Figure 3E). The results of this genetic epistasis analysis suggest that Rif2 and Rpd3L may function together in a common pathway to promote a telomere fold-back.

To mechanistically understand the genetic relationships between the Rpd3L complex and Rif2, we performed ChIP experiments to determine if the *rif2Δ* mutation had an effect on Rpd3L (Rxt2-TAP) localization at telomeres. Subtelomeric DNA was enriched above non-tagged wild type control cells (background) with an epitope-tagged Rxt2 allele both close to (-6 bp) (Figure 3F) and up to 2000 base pairs away from the telomeric tracts (Figure 3F) as previously described [18]. This enrichment was decreased to near background levels in a *rif2Δ* mutant (Figure 3F). The loss of ChIP signal is not due to altered expression levels of Rxt2-TAP in *rif2Δ* mutants as confirmed by western blot analysis (Figure S3B). Unlike Rpd3L, the ability to cross-link the Rpd3S complex (Rco1-TAP) to subtelomeric regions was not altered in *rif2Δ* cells (Figure 3G and Figure S3C). Together these data suggest that the Rif2 promotes a structural alteration at telomeres through the recruitment of the Rpd3L KDAC complex. Moreover, the Hda1 KDAC as well as the Rpd3S complex promote the same fold-back structure, but independent of the Rif2/Rpd3L pathway.

### The telomere fold-back structure may have protective functions

**Figure 4:**
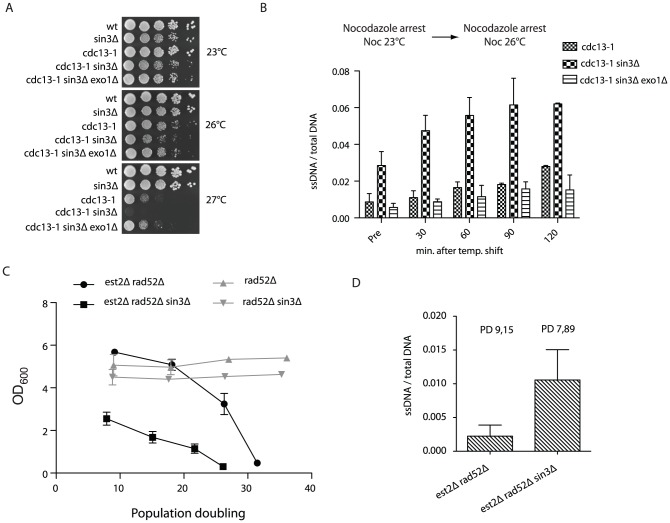
Rpd3 promotes telomere end protection. (A) Strains with the indicated genotypes were spotted onto YPD media following an overnight culture at 23°C and incubated at various temperatures for 3–4 days before being imaged. (B) *cdc13-1*, *cdc13-1 sin3Δ* and *cdc13-1 sin3Δ exo1Δ* cells were arrested in nocodazole at 23°C for three hours before being shifted to 26°C, after which DNA was extracted at 30 minute intervals. Non-denatured and denatured DNA was dot blotted onto a membrane and incubated with a DIG-labeled probe (oBL 207) to recognize the telomeric 3′ ssDNA overhang. The amount of ssDNA is represented as non-denatured DNA as a fraction of the total (as determined by the amount of denatured telomeric DNA). Error bars represent SD from three independent experiments. Pre = before the 26°C temperature shift. (C) The indicated genotypes were derived via tetrad dissection of the heterozygous diploid strain (yMD 1146) and diluted to an OD_600_ 0.01. Cells were grown for 24-hour intervals before being measured and re-diluted. The rate of senescence was increased in *est2Δ rad52Δ* cells when *SIN3* was subsequently deleted, n = 8 for each genotype. The growth rates of *rad52Δ* (n = 3) and *rad52Δ sin3Δ* (n = 3) were similar. Population doubling refers to the number of doublings post spore germination. (D) Genomic DNA was isolated in both non-denaturing and denaturing conditions from the indicated genotypes (each n = 3) using samples generated in (C) at the specified population doubling (PD). Single-stranded telomeric DNA was detected upon hybridization with DIG labeled oBL 207 and normalized to total telomeric DNA following a denaturation step.

To better understand the function of the loop structure and whether or not it may have a protective role at the telomere we impaired looping (deletion of *SIN3*) in various genetic backgrounds where telomere function was compromised. In *cdc13-1 sin3Δ* double mutants we observed a temperature-dependent synthetic lethality in the double mutant, compared to the respective single mutants (Figure 4A), indicating that partially uncapped telomeres (*cdc13-1*) may require a Rpd3-mediated structure for viability. The negative genetic interaction was suppressed by the further deletion of *EXO1*, the nuclease responsible for the majority of telomere resection at dysfunctional telomeres (Figure 4A). To ensure that this interaction was a direct consequence of telomere dysfunction, we assayed the accumulation of telomeric ssDNA following the shift of nocodazole-arrested *cdc13-1* and *cdc13-1 sin3Δ* cells from 23°C to the semi-permissive temperature of 26°C (Figure 4B). In agreement with the negative genetic interaction (Figure 4A), we observed an Exo1 dependent increase in telomeric ssDNA in the double mutant above that seen in the *cdc13-1* single mutant (Figure 4B). Importantly, we did not detect an increase in ssDNA at telomeres in *sin3Δ* single mutants when compared to isogenic wild type control cells (Figure S4A).

To determine if Rpd3 dependent telomere structure may have an influence on the rate of cellular senescence, we compared senescence onset in both *est2Δ rad52Δ* (where HR and telomerase-mediated telomere elongation are impaired) and *est2Δ rad52Δ sin3Δ* mutants. The deletion of *SIN3* resulted in a dramatic increase in the rate of cellular senescence in the absence of telomerase and homologous recombination (Figure 4C). The accelerated loss of viability associated with the *sin3Δ* mutation is specifically related to the absence of telomerase as *rad52Δ* cells maintain viability to a similar extent as *rad52Δ sin3Δ* cells (Figure 4C). To better understand the cause of premature senescence in *sin3Δ* mutants, genomic DNA was prepared from the senescence curves (Figure 4C) and both single stranded telomeric DNA accumulation and telomere length were analyzed. As is the case when combined with the *cdc13-1* mutant, we found that *sin3Δ est2Δ rad52Δ* mutants had increased levels of ssDNA at telomeres compared to isogenic *est2Δ rad52Δ* cells (Figure 4D). The telomere shortening rate is unaffected between *sin3Δ est2Δ rad52Δ* and *est2Δ rad52Δ* strains (Figure S4B, S4C), however there is evidence of early rapid telomere loss events at some telomeres in the triple mutant compared to the isogenic double (Figure S4B).

Taken together these results imply that the Rpd3 lysine deacetylase is essential to prevent excessive nuclease-mediated resection specifically at uncapped telomeres. In the absence of a telomere lengthening mechanism this resection may lead to excessive telomere shortening. We propose that this protective function may involve the formation of a fold-back structure at the chromosome ends.

## Discussion

We have demonstrated that multiple biological processes influence the ability of telomeres to form a higher-order fold-back structure. High-throughput screening coupled to stringent bioinformatic analysis, has revealed that class I (Rpd3) and class II (Hda1) KDAC activities were among the most significantly enriched biological processes required to promote the formation/maintenance of a telomere loop. In addition, the Rap1 binding proteins, Rif1 and Rif2, which localize directly to telomeres, were implicated in telomere fold-back establishment. Through genetic epistasis analysis, we found that *rif2Δ* and mutants of the Rpd3L complex did not have additive structural defects at telomeres, suggesting that they may function together in a single pathway. This genetic interaction was confirmed by demonstrating that Rpd3L was no longer able to localize to telomeres in *rif2Δ* cells. Furthermore, we have shown that the telomeres in mutants with looping defects are more susceptible to uncapping, nucleolytic degradation, telomere loss and promote accelerated rates of cellular senescence in the absence of telomere maintenance. A recent study has reported that the Rpd3 complex is required to prevent chromosome end fusions in *Drosophila melanogaster* [37]. Together, these data suggest that the regulation of such telomere fold-back structures may be conserved, and furthermore indicate that multiple cellular processes, apart from those that directly impinge on DNA metabolism, may have effects on telomere structure/function.

**Figure 5:**
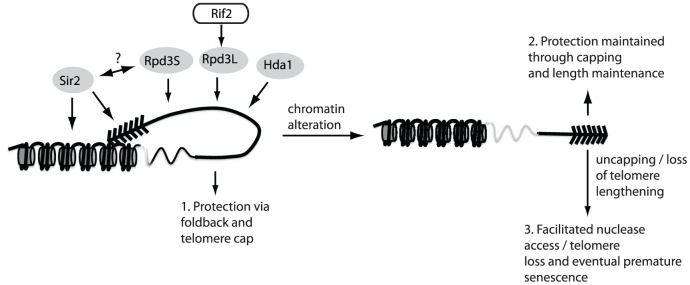
The fold-back may contribute to telomere protection. The maintenance of telomere structure requires the telomere-bound Rif2 protein to ensure that the Rpd3L complex gets properly loaded/maintained at chromosome ends. The presence of the Rpd3L KDAC (as well as Rpd3S, Sir2 and Hda1) promotes a protective structure at telomeres, which likely eminates in a fold-back of the telomeric DNA onto the subtelomeric region (1.). In the absence of this structure, telomeres remain protected due to a combination of telomerase-mediated elongation and capping via the CST complex (2.). When both capping and the fold-back structure are simultaneously compromised (3.) chromosome ends undergo accelerated nucleolytic degradation, and experience an accelerated rate of senescence in cells lacking a telomere maintenance mechanism due to the fact that rapidly resected uncapped telomeres do not get re-elongated.

Although we have focused on the telomere dysfunction phenotypes associated *rpd3* mutants, it is of interest that many of the other mutants recovered in our telomere structure screen have been previously identified to have negative synthetic interactions with *cdc13-1* (*rrd1Δ, swr1Δ, arp6Δ, spe1Δ, spe3Δ, oca1Δ, oca2Δ, oca5Δ, elp4Δ, pep8Δ, rif1Δ, yme1Δ, htd2Δ, ski3Δ, rim101Δ, ede1Δ*) [10], [38]–[39] as well as an increased rate of replicative senescence (*hda1Δ, sin3Δ, sec28Δ, med1Δ, asf1Δ, rif1Δ, rif2Δ, arp6Δ, elp4Δ*) [40]–[41] (see Table 1 for a complete overview of overlaps between our screen and other selected telomere function screens). Moreover, the Sir2/3/4 complex, which is required to form a telomere fold-back structure [29] also prevents premature senescence [42]. This overlap between our screen and previously published data suggests that telomere structure may make significant contributions towards preserving telomere integrity when telomere function is compromised (Figure 5). Consistently, *sin3Δ* single mutants do not exhibit increased ssDNA accumulation at telomeres nor do they have any changes in telomere length in comparison to isogenic wild type strains. This would suggest that in the absence of a telomere fold-back, the CST complex and other capping factors are sufficient to maintain a protected state (Figure 5). However, when CST function is compromised (e.g. *cdc13-1*) in combination with an inability to fold-back, resection becomes accelerated (Figure 4B, 5). In terms of telomere-induced cellular senescence in the absence of telomere maintenance, the increased resection in non-looped mutants would also lead to increased telomere shortening (Figure 5). It will be of interest to perform an extensive genetic epistasis of all mutants isolated in the loop screen in order to determine if the negative interactions with *cdc13-1* are epistatic (i.e. due to a fold-back defect).

There was also a large overlap between mutants found in our screen and mutants that have been implicated in both the positive and negative regulation of telomere length (*vps28Δ, trk1Δ, ctk1Δ, mrt4Δ, rif1Δ, sur4Δ, nut1Δ, siw14Δ, leo1Δ, hit1Δ, pcp1Δ, pdx3Δ*) [43]–[44]. This would suggest that either telomere looping has a direct effect on telomere length regulation, or conversely, may indicate that telomere length changes impinge on the ability to form a fold-back. Our results suggest that telomere looping does not affect telomere length homeostasis directly, as many of the mutants recovered in our screen, even those with “strong” looping defects, have wild type telomere length. On the other hand, telomere length changes could indeed have a drastic effect on telomere structure in terms of the chromatin alterations that occur with respect to length changes. Telomere shortening, for example, results in de-silencing in the subtelomeric region [45] due to the decreased capacity of shortened telomeres to recruit the Sir2/3/4 histone deacetylase complex, which is required for loop formation in yeast [29]. Long telomeres, in contrast, promote a hyper-silenced state in the subtelomere [45], much like what occurs in *rif1Δ* and *rif2Δ* mutants, where, in the case of the latter mutant, Rpd3L fails to localize to telomeres. Indeed, Rpd3 mutants (L and S) are hypersilenced in the subtelomeric zone. One possibility would be that long telomeres (as seen in *rif2Δ*) mutants fail to properly localize Rpd3L, which leads to a subsequent fold-back defect. Although our screen implicates proper length regulation as a key regulator of telomere looping, they remain correlative and require further investigation in order to draw concrete conclusions.

Whereas both the Rpd3S and Hda1 complexes were additive with *rif2Δ* mutants in terms of a looping defect, the Rpd3L complex was epistatic. This relationship was confirmed mechanistically as we noticed that Rpd3L is not able to properly localize to telomeres in *rif2Δ* cells. These epistasis analyses revealed that multiple KDACs contribute to telomere looping (Figure 5). Rpd3L, Rpd3S and Hda1 all promote telomere looping in parallel pathways whereas the relationship between the Sir2/3/4 complex and the other KDACs in terms of telomere structure remains enigmatic. The KDACs are best known for their deacetylation of histones and they are known to contribute significantly to silencing at subtelomeric loci [18]. Consistent with a connection between chromatin modification and the telomere fold-back structure, we also found that many members of the Swr1 chromatin remodeling complex as well as the histone chaperone Asf1 (Table 1) were important for telomere folding. A future challenge will be to determine the targets of the KDACs. Although subtelomeric histones are prime candidates, telomeric proteins themselves may be targets. Furthermore, it will be important to characterize how the other mutants revealed in the screen contribute to telomere looping and to understand if these mutants are epistatic with the KDACs. Indeed, it has been difficult to understand why mutations that affect such diverse biological pathways may have synthetic growth defects with *cdc13-1* or in some cases, senesce rapidly [38]–[39], [41]. Since many signaling pathways activate effectors via activation/repression of target genes through chromatin remodeling and/or histone acetylation/deacetylation, we propose that activation or repression of these pathways may influence the ability of the KDACS (Rpd3/Hda1 and Sir2) to act at telomeres and in turn directly or indirectly influence telomere structure.

This work has uncovered multiple regulators of the telomere fold-back structure, including lysine deactylation and chromatin remodeling. The results of our screen correlate well with screens that have been performed to elucidate genes implicated in telomere function and cellular senescence suggesting that the fold-back structure may be important for chromosome end protection. Previous models have speculated that the fold-back in yeast may be important to establish silent chromatin within the telomeric/subtelomeric loci [46]–[47]. As an alternative, it was also suggested that silent telomeric chromatin may be required to establish a particular architecture that contributes to chromosome end protection [47]. Our results indicate that silent chromatin can be established in the absence of a telomere fold-back since many of the mutants recovered in our screen are not compromised for silencing (Table 1) or even have slightly enhanced silencing (e.g. *rpd3* mutants). Sir2/3/4-mediated silencing as well as other chromatin modifications are however important to establish a telomere loop, which likely promotes end protection. Interestingly, Rpd3 dependent histone deacetylation has been shown to prevent Sir2/3/4 protein spreading towards the centromere [17], which may potentially deplete SIR protein levels immediately adjacent to the telomere, raising the possibility that *SIR2/3/4* disruption and *RPD3L/S* disruption may be one in the same in terms of a fold-back defect. Further characterization of the yeast fold-back structure and the relationship to silent chromatin will be essential in order to clarify these issues. In summary, by understanding how different biological processes impinge on chromosome end structure, we increase the possibilities to manipulate telomere function, both positively and negatively, which may have important implications for diseases that stem from telomere dysfunction.

## Materials and Methods

### Yeast culturing and strains

Standard yeast media and growth conditions were used [48]. Yeast strains used in this study are listed in the Table S1.

### Yeast spot dilutions

For spotting assays, yeast cells were incubated overnight at appropriate temperature in YPD. Cells were diluted to OD_600_ 0.5 and spotted in ten-fold dilutions onto 2% raffinose, 1% galactose plates either with (+FOA) or without (−FOA) 5-FOA. Cells were incubated for 3 days at proper temperature, imaged and then replica plated on SD-URA plates for 2 days at the same temperature before imaging.

### Telomere PCR

Telomere PCR was performed using 100 ng genomic DNA diluted in 1× NEB4 buffer and water. Samples were denatured for 10 min at 96°C and cooled to 4°C. Tailing mix (4 U/µl terminal transferase (NEB), 1× NEB4 buffer, 1 mM CTPs) was added to a final concentration of 10%. Tailing reaction was performed as the follows: 37°C 30 min, 65°C 10 min, 96°C 5 min, arrest at 65°C. 3× volume of preheated PCR-MIX (1 µM oligo dG reverse primer, 1 µM telomere specific forward primer either 1L, 6R, 7L or Y′, 0.267 mM dNTPs, 0.083 U/µl Phusion polymerase (NEB), PCR buffer (89.11 mM Tris-HCl pH 8.8, 21.28 mM (NH_4_)_2_SO_4_, 6.65% glycerol, 0.0133% Tween-20) was added and PCR reaction was performed using: 95°C 3 min, 45 cycles: (95°C 30 s, 68°C 15 s, 72°C 20 s), 68°C 5 min, hold on 12°C.

Samples were mixed with DNA loading buffer and separated on a 1.8% agarose gel for 30 min at 100 V. Bands were detected using LAS-4000 (Fujifilm) and quantified using Multi Gauge Software (Fujifilm). A complete lis of oligonucleotides used in this study can be found in Table S2.

### Senescence curves

Spore-colonies of dissected heterozygous diploids were suspended in water and diluted in 5 ml YPD medium to a final concentration of OD_600_ 0.01. Cells were incubated for 24 h at 30°C and absorption at 600 nm was measured. Cultures were re-diluted to OD_600_ 0.01 in 5 ml YPD and inoculated for further 24 h at 30°C. Each day cell samples were harvested and genomic DNA was prepared for telomere length analysis (Quiagen genomic DNA prep. Kit). Population doublings (PD) were calculated as log_2_(OD_600_^24 h^/0.01). All PD values refer to PD after the spore colony had been harvested from the dissection plate (about 25 generations). Graphs were made in Prism5 (GraphPad).

### High-throughput screening

Synthetic Genetic Array (SGA) methodology was used (strains R1459 and R1460) to obtain haploid gene deletion mutants containing either construct 1A or construct 2 (Tong and Boone, 2006). Cells were then replica-pinned onto media containing galactose, with and without 5-FOA. Growth on 5-FOA was compared between construct 2-containing deletion mutants and construct 1-containing mutants (comparison 1). Growth of construct 2-containing mutants was also compared with and without the presence of 5-FOA (comparison 2). Construct 2-containing mutants that grew better by either comparison 1 or 2 were selected for validation. Validation was carried out by manually crossing and dissecting tetrads from independent starter strains followed by duplicate spot assays onto media with and without 5-FOA.

### Chromatin immunoprecipitation

Yeast cells were grown over night at 30°C and diluted to OD600 0.2. They were grown until exp. Phase (OD600 0.6–1.0), crosslinked for 8 min (20 min for Rxt2-TAP-ChIP, 10 min for Cdc13-TAP-Chip) with formaldehyde (final conc. 1.2%) and quenched with glycine (360 mM final). After adjusting the volume to the same OD all samples were washed two times with 1× PBS, resuspended in FA Lysis Buffer (50 mM HEPES-KOH pH 7.5, 140 mM NaCl, 1 mM EDTA pH 8, 1% Triton X-100, 0.1% Sodium deoxycholate) and lysed with Matrix C tubes via FastPrep (6.5 M/s, 2°—30 sec with 1 min break). Cell extracts were recovered, centrifuged and the soluble potion of the lysate was discarded. Pellets were resuspended in FA buffer +SDS (2% final) and split up for sonication. Chromatin was sheared 30 sec on/off for 15 min. Supernantant (ChIP extract) was diluted to 1 mg/ml protein concentration in FA buffer and used for immunoprecipitation (IP).

Pre incubated protein G sepharose beads (washed with 1×PBS, FA Buffer and pre-incubated with 5% BSA for 1 h at 4°C) were added to the 1 mg/ml solution to perform an addition precleaning step before the IP (1 h at 4°C). After precleaning anti-Rap1 antibody (Santa Cruz) was added to the solution (1∶100) and incubated with fresh beads over night at 4°C, rotating.

For Tap-ChIPs (Rxt2 and Cdc13) IgG-Sepharose Beads (washed with 1× PBS and FA-buffer) were added to the 1 mg/ml solution and IP was incubated over night at 4°C.

Sonication efficiency was tested via cleaning 100 µl of the ChIP extract and performing agarose gelelectrophoresis. IP was washed with FA-Lysis buffer, FALysis buffer 500 (FA buffer with 500 mM NaCl), Buffer3 (10 mM Tris-HCl pH 8 1 mM EDTA pH 8, 250 mM LiCl, 1%NP-40, 1% Sodium deoxycholate), and TE (pH 8). For elution buffer B (50 mM Tris-HCl ph 7.5, 1% SDS, 10 mM EDTA pH 8) was added and IP was incubated at 65°C for 8 mins. For reverse-crosslinking proteinase K was added to the IP and INPUT control (ChIP extract, 1 mg/ml solution without IP) and incubated at 65°C, rotating overnight. Samples were cleaned with Quiagen “QIAquick PCR Purification Kit” and qPCR analysis was performed using Roche standard PCR protocol for Sybr-Green detection with 55°C annealing temperature, all oligonucleotides used are listen in Table S2. Measured ct values were corrected to INPUT and normalized to the actin signal using the following formulas.

Rap1 ChIP:
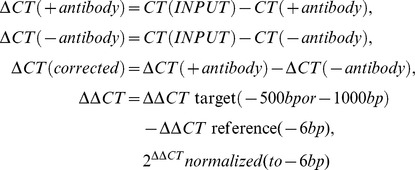
Rxt2-TAP ChIP:
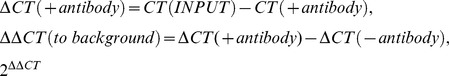
Cdc13-TAP ChIP:
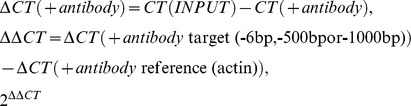



### Cell cycle arrest and ssDNA dot blotting

Cells were grown overnight at 23°C in 10 ml YPD. Saturated cultures were diluted to OD_600_ 0.2 in 150 ml YPD and incubated at 23°C until they reach log phase (0.6–0.8). Nocodazol (20 µg/ml final) was added and cells were incubated for a further 3 h at 23°C, shaking. Cells were checked under the microscope until >90% were largebudded. “Pre” samples were harvested and cells were subsequently shifted to 26°C.

Additional samples were collected for all time points (30 min, 60 min, 90 min and 120 min) after the shift. For ssDNA analysis, dot blotting was performed. DNA was extracted using genomic DNA Kit (Quiagen). Isolated DNA was either denatured using 0.2 M NaOH and 65°C for 15 min or kept on ice for native conditions.

For blotting, 4 µg DNA (native) or 0.5 µg (denatured) were suspended in 200 µl 2×SSC and loaded to the dot blot apparatus using nylon membrane (GE Healthcare Amersham H-bond). After crosslinking (UV Stratalinker 2400, Stratagene) DIG labeling (DIG labeled probe oBL207) and detection was performed as described by the product guidelines (Roche DIG oligonucleotide 3′labeling KIT).

### URA3 induction and Northern blotting

Cells were grown overnight at 30°C in 5 ml SD medium containing 2% Raffinose (S-Raf). Saturated cultures were diluted to OD_600_ 0.2 in 8 ml S-Raf and incubated at 30°C until they reach log phase (0.6–0.8). Cells were split and 2% galactose or 2% glucose (final) were added. Cells were incubated for 2 1/2 h at 30°C, shaking. Cells were centrifuged down and RNA was extracted and Northern Blotting was performed as described previously [42]. URA3 and actin were detected using DiG labeled PCR products (Roche, “DiG High Prime” labeling) gained from a PCR reaction with oBL17, oBL18 for URA3 and oBL292, oBL293 for actin (1. 98°C 30 sec, 2. 98°C 10 sec, 3. 60°C 30 sec, 4. 72°C 1 min, 5. 72°C 5 min, 12°C forever, repeating steps 2–4 for 33 cycles). Quantification was performed using Multi Gauge software (Fujiifilm) and signal was displayed as URA3 over actin.

### Protein extraction and Western blotting

3 ml of culture (log phase) was centrifuged at 13000 rpm for 2 min. Pellets were resuspended with 150 µl solution 1 (0,97 M β-mercaptoethanol, 1,8 M NaOH) and incubated on ice for 10 min. 150 µl, 50% TCA was added and cells were incubated 10 min on ice, centrifuged at 13000 rpm for 2 min at 4°C and the pellet was resuspended with 1 ml acetone. Solution was centrifuged at 13000 rpm for 2 min at 4°C and the pellet was resuspended in 140 µl UREA buffer (120 mM Tris-HCL pH 6,8, 5% Glycerol final, 8 M Urea final, 143 M 2-mercaptoethanol final, 8% SDS final, a little bit of bromphenol blue indicator). Protein extract was incubated 5 min at 95°C, centrifuged and loaded on a pre-cast gradient Gel (BioRad).

## Supporting Information

Figure S1 A) Examples of the different qualitative assignments given to looping mutants from the screen where +++ = strong defect, ++ = moderate and + = weak, the mutant ORFs are indicated. B), C) The indicated mutants were grown for over 100 generations and subject to telomere PCR at telomere 1L and at Y′ telomeres. Telomere lengths are represented with respect to wild type (which is set to 0). *rif2Δ* and *est2Δ* mutants serve as controls for mutants with long and short telomeres, respectively. D) Northern blot quantification of URA3 expression in strain harboring construct 2. Strains were either grown in the presents of glucose (uninduced) or galactose (induced). RNA was isolated and northern blot bands were quantified using Multi Gauge software (FujiiFilm). Signal was calculated as *URA3* RNA over actin RNA.
(EPS)

Figure S2 Sonication control for the Rap1-ChIP; RAP1 and Cdc13-TAP protein expression. A) DNA-sonication control of the chromatin fragments for Rap1-ChIP (*hda1*Δ and *sin3*Δ). All fragments have an average bulk size of 300 bp. B) Western blot for Rap1 total protein level in *hda1*Δ and *sin3*Δ or *sir4*Δ mutants reveals no differences in general RAP1 expression. Rap1-TAP causes a shift in the Rap1 protein band due to the TAP-tag. C) Western blot for Cdc13-TAP protein level in wild type or *sin3*Δ cells. Tap-tag was detected via peroxidase-anti-peroxidase antibody. D and E) Telomere PCR at telomere 1L revealed a shortening upon *EST2* disruption following 25 and 50 generations of growth. F) Enrichment of the -6 position at natural telomere 6R is increased upon telomere shortening following ChIP directed against Cdc13-TAP.
(EPS)

Figure S3 Rap1, Rxt2-TAP- and Rco1-TAP protein expression. A), B) and C) Western blot analysis of Rap1, Rxt2-TAP and Rco1-TAP protein levels. Protein was extracted from cells with the indicated genotypes and western blotting was performed with either an anti-Rap1 antibody (A) or a mouse, anti-actin antibody which through its Fc domain cross reacts with the TAP epitope tag on Rxt2-TAP and Rco1-TAP respectively (B). Expression levels of Rap1, Rxt2-TAP and Rco1-TAP do not differ when comparing a wild type background with a *rif2Δ* (and *rif1Δ*) background.
(EPS)

Figure S4 ssDNA analysis of *sin3*Δ mutants and telomere length analysis for senescence curves on telomere 6R, 7L and Y′telomeres. A) Cells of the indicated genotype were arrested in nocodazole for 2.5 hours before genomic DNA was extracted and dot blotted in native and denaturing conditions. Quantification of a dot blot analysis where the amount of ss telomeric DNA is normalized to total telomeric DNA. B) Telomere PCR for telomere 6R, and Y′ telomeres to follow the rate of telomere shortening from the survivor curves described in Figure 4C.
(EPS)

Table S1 Yeast strains used in this study. All strains used in this study were derived from BY4741 background (*his3-1, leu2-0, ura3-0, met1-0*) unless indicated otherwise.
(PDF)

Table S2 Oligonucleotides used in this study. All oligos used in this study are listed and are in the 5′to 3′ (left to right) direction.
(PDF)
