# Supplementary material for: Rif2 Promotes a Telomere Fold-Back Structure through Rpd3L Recruitment in Budding Yeast
Source: PLoS Genet. 2012 Sep 20;8(9):e1002960. doi: 10.1371/journal.pgen.1002960 (PMC3447961; doi:10.1371/journal.pgen.1002960)
Supplement: Table S1 — Yeast strains used in this study. All strains used in this study were derived from BY4741 background (his3-1, leu2-0, ura3-0, met1-0) unless indicated otherwise. (PDF) [file pgen.1002960.s005.pdf]

Table S1 – yeast strains used in this study

Yeast strains used in this study were derived from BY4741 background (*his3-1, leu2-0, ura3-0, met1-0*)

Code	Name	Genotype
YHP88	wild type con2	Mat**a** *gal80::NAT 7L::Con2-URA3*
YHP77	rif1 con2	Mat**a** *rif1::KAN gal80::NAT 7L::Con2-URA3*
YHP81	rif2 con2	Mat**a** *rif2::KAN gal80::NAT 7L::Con2-URA3*
YHP241	cdc13 exo1 con2	Mat**a** *cdc13::KAN exo1::HIS gal80::NAT 7L::Con2-URA3*
YHP251	exo1 con2	Mat**a** *exo1::HIS gal80::NAT 7L::Con2-URA3*
YHP252	stn1 exo1 con2	Mat**α** *stn1::KAN exo1::HIS3 gal80::NAT 7L::Con2-URA3*
YHP121	wild type con4	Mat**a** *gal80::NAT 7L::Con4-URA3*
YHP123	rif1 con4	Mat**a** *gal80::NAT rif1::KAN 7L::Con4-URA3*
YHP125	rif2 con4	Mat**a** *gal80::NAT rif2::KAN 7L::Con4-URA3*
YHP225	rif2	Mat**a** *rif2::KAN*
YHP224	sir4	Mat**α** *sir4::HIS*
YHP230	sir4 rif2	Mat**α** *sir4::HIS rif2::KAN*
YHP74	wild type con1A	Mat**α** *gal80::NAT 7L::Con1A-URA3*
YHP66	rif1 con1A	Mat**a** *rif1::KAN gal80::NAT 7L::Con1A-URA3*
YHP71	rif2 con1A	Mat**a** *rif2::KAN gal80::NAT 7L::Con1A-URA3*
YHP155	rif1 rif2 con1A	Mat**α** *rif1::KAN rif2::HIS3 gal80::NAT 7L::Con1A-URA3*
YHP157	rif1 rif2 con2	Mat**α** *rif1::KAN rif2::HIS3 gal80::NAT 7L::Con2-URA3*
YHP297	rif2 est2 con2	Mat**a** *rif2::KAN est2::HIS3 gal80::NAT 7L::Con2-URA3*
YHP305	cdc13 exo1 est2 con2	Mat**a** *cdc13::cdc13-1KAN exo1::HIS3 est2::HYG gal80::NAT 7L::Con2-URA3*
YHP302	stn1 exo1 est2 con2	Mat**α** *stn1::stn1-13KAN exo1::HIS3 est2::HYG gal80::NAT 7L::Con2-URA3*
YHP273	stn1 exo1 rif2 con2	Mat**a** *stn1::stn1-13KAN exo1::HIS3 rif2::KAN gal80::NAT 7L::Con2-URA3*
YHP275	cdc13 exo1 rif2 con2	Mat**a** *cdc13::cdc13-1KAN exo1::HIS3 rif2::KAN gal80::NAT 7L::Con2-URA3*
YHP299	est2 con2	Mat**a** *est2::HYG gal80::NAT 7L::Con2-URA3*
YMD527	rxt2	Mat**a** *rxt2::KAN*
YMD30	sap30	Mat**a** *sap30::KAN*
YMD533	rpd3	Mat**a** *rpd3::KAN*

YMD195	sin3	Mat**a** *sin3::KAN*
YMD132	eaf3	Mat**a** *eaf3::KAN*
YMD186	rco1	Mat**a** *rco1::KAN*
YHP313	Rxt2-TAP	Mat**a** *rxt2*::RXT2-TAP marked with HIS
YHP315	Rxt2-TAP rif2	Matα *rxt2*::RXT2-TAP marked with HIS *rif2::KAN*
YJK56	sin3	Matα *sin3::NAT*
YJK55	cdc13-1	Mat**a** *cdc13::cdc13-1KAN*
YJK57	cdc13-1 sin3	Mat**a** *cdc13::cdc13-1KAN sin3::NAT*
YBL725	tel2-7 con2	Matα *tel2::tel2-7KAN gal80::NAT 7L::Con2-URA3*
YBL727	stn1-13 con2	Mat**a** *stn1::stn1-13KAN gal80::NAT 7L::Con2-URA3*
YBL729	cdc13-1 con2	Matα *cdc13::cdc13-1KAN gal80::NAT 7L::Con2-URA3*
YBL721	rap1-1 con2	Mat**a** *rap1::rap1-1KAN gal80::NAT 7L::Con2-URA3*
YBL723	rap1-2 con2	Mat**a** *rap1::rap1-2KAN gal80::NAT 7L::Con2-URA3*
YHP266	cdc13-1 exo1 con1A	Matα *cdc13::cdc13-1KAN exo1::HIS3 gal80::NAT 7L::Con1A-URA3*
YHP260	stn1-13 exo1 con1A	Mat**a** *stn1::stn1-13KAN exo1::HIS3 gal80::NAT 7L::Con1A-URA3*
YHP214	rif1 est2 con1A	Mat**a** *rif1::KAN est2::HIS3 gal80::NAT 7L::Con1A-URA3*
YHP215	rif2 est2 con1A	Mat**a** *rif2::KAN est2::HIS3 gal80::NAT 7L::Con1A-URA3*
YHP307	cdc13-1 exo1 est2 con1A	Matα *cdc13::cdc13-1KAN exo1::HIS3 est2::HYG gal80::NAT 7L::Con1A-URA3*
YHP303	stn1-13 exo1 est2 con1A	Mat**a** *stn1::stn1-13KAN exo1::HIS3 est2::HYG gal80::NAT 7L::Con1A-URA3*
YHP296	rif1 est2 con2	Mat**a** *rif1::KAN est2::HIS3 gal80::NAT 7L::Con2-URA3*
YMD923	wild type con4	Mat**a** *gal80::NAT 7L::Con4-URA3*
YMD924	rxt2 con4	Matα *rxt2::KAN gal80::NAT 7L::Con4-URA3*
YMD925	sds3 con4	Mat**a** *sds3::KAN gal80::NAT 7L::Con4-URA3*
YMD926	pho23 con4	Matα *pho23::KAN gal80::NAT 7L::Con4-URA3*
YMP927	sap30 con4	Mat**a** *sap30::KAN gal80::NAT 7L::Con4-URA3*
YMD928	sin3 con4	Matα *sin3::KAN gal80::NAT 7L::Con4-URA3*
YMD929	rco1 con4	Mat**a** *rco1::KAN gal80::NAT 7L::Con4-URA3*
YMD930	eaf1 con4	Matα *eaf1::KAN gal80::NAT 7L::Con4-URA3*
YMD1022	hda1 con4	Mat**a** *hda1::KAN gal80::NAT 7L::Con4-URA3*
YMD1026	hda2 con4	Mat**a** *hda2::KAN gal80::NAT 7L::Con4-URA3*
YMD1030	hda3 con4	Mat**a** *hda3::KAN gal80::NAT 7L::Con4-URA3*
YMD10	hda1	Mat**a** *hda1::KAN*

YMD203	hda2	Mat**a** *hda2::KAN*
YMD181	hda3	Mat**a** *hda3::KAN*
YMD967	rxt2 con2	Matα *rxt2::KAN gal80::NAT 7L::Con2-URA3*
YMD969	rxt2 rif2 con2	Mat**a** *rxt2::KAN rif2::HYG gal80::NAT 7L::Con2-URA3*
YMD995	sap30 con2	Mat**a** *sap30::KAN gal80::NAT 7L::Con2-URA3*
YMD997	sap30 rif2 con2	Matα *sap30::KAN rif2::HYG gal80::NAT 7L::Con2-URA3*
YMD1164	rad52 est2	Mat**a** *rad52::NAT est2::HIS3*
YMD1169	rad52 est2 sin3	Mat**a** *rad52::NAT est2::HIS3 sin3::KAN*
YMD975	sin3 con2	Mat**a** *sin3::KAN gal80::NAT 7L::Con2-URA3*
YMD977	sin3 rif2 con2	Matα *sin3::KAN rif2::HYG gal80::NAT 7L::Con2-URA3*
YMD960	rco1 con2	Mat**a** *rco1::KAN gal80::NAT 7L::Con2-URA3*
YMD962	rco1 rif2 con2	Matα *rco1::KAN rif2::HYG gal80::NAT 7L::Con2-URA3*
YMD987	eaf3 con2	Mat**a** *eaf3::KAN gal80::NAT 7L::Con2-URA3*
YMD989	eaf3 rif2 con2	Mat**a** *eaf3::KAN rif2::HYG gal80::NAT 7L::Con2-URA3*
YMD1110	hda1 con2	Matα *hda1::KAN gal80::NAT 7L::Con2-URA3*
YMD1112	hda1 rif2 con2	Mat**a** *hda1::KAN rif2::HYG gal80::NAT 7L::Con2-URA3*
YMD1113	hda2 con2	Mat**a** *hda2::KAN gal80::NAT 7L::Con2-URA3*
YMD1116	hda2 rif2 con2	Mat**a** *hda2::KAN rif2::HYG gal80::NAT 7L::Con2-URA3*
YMD1131	rxt2 eaf3 con2	Mat**a** *eaf3::KAN rxt2::HIS3 gal80::NAT 7L::Con2-URA3*
YMD1133	rxt2 eaf3 rif2 con2	Matα *eaf3::KAN rxt2::HIS3 rif2::HYG gal80::NAT 7L::Con2-URA3*
YMD911	sds3 con2	Mat**a** *sds3::KAN gal80::NAT 7L::Con2-URA3*
YMD291	sap30 con2	Mat**a** *sap30::KAN gal80::NAT 7L::Con2-URA3*
YMD241	hda3 con2	Mat**a** *hda3::KAN gal80::NAT 7L::Con2-URA3*
YMD299	pho23 con2	Mat**a** *pho23::KAN gal80::NAT 7L::Con2-URA3*
YMD415	YHL029C con2	Mat**a** *YHL029C::KAN gal80::NAT 7L::Con2-URA3*
YMD281	YBR103W con2	Mat**a** *YBR103W::KAN gal80::NAT 7L::Con2-URA3*
YMD255	YBR275C con2	Mat**a** *YBR275C::KAN gal80::NAT 7L::Con2-URA3*
YMD253	YPR070W con2	Mat**a** *YPR070W::KAN gal80::NAT 7L::Con2-URA3*
YMD251	YML035C con2	Mat**a** *YML035C::KAN gal80::NAT 7L::Con2-URA3*
YHP415	Cdc13-TAP	Matα *Cdc13-TAP marked with HIS3*
YHP417	Cdc13-TAP sin3	Mat**a** *Cdc13-TAP marked with HIS3 sin3::KAN*
YMD78	rif1	Mat**a** *rif1::KAN*
YHP314	Rco1-TAP	Mat**a** *Rco1-TAP marked with HIS3*

YHP319	Rco1-TAP rif2	Mat**α** *Rco1-TAP marked with HIS3 rif2::KAN*
YHP326	cdc13-1 sin3 exo1	Mat**a** *cdc13-1::KAN sin3::NAT exo1::HIS3*
YBL259	Rap1-TAP	Mat**a** *Rap1-TAP marked with HIS3*
YHP373	rad52	Mat**a** *rad52::NAT*
YMD1146	rad52 sin3 est2	Mat**a**/Mat**α** Het. *rad52::NAT sin3::KAN est2::HIS3*
YHP379	rad52 sin3	Mat**a** *rad52::NAT sin3::KAN*
