# Supplementary material for: Rif2 Promotes a Telomere Fold-Back Structure through Rpd3L Recruitment in Budding Yeast
Source: PLoS Genet. 2012 Sep 20;8(9):e1002960. doi: 10.1371/journal.pgen.1002960 (PMC3447961; doi:10.1371/journal.pgen.1002960)
Supplement: Table S2 — Oligonucleotides used in this study. All oligos used in this study are listed and are in the 5′to 3′ (left to right) direction. (PDF) [file pgen.1002960.s006.pdf]

Table S2 – oligonucleotides used in this study

Code	Name	Sequence
oBL359	oligo dG reverse primer	CGG GAT CC(G)$_{18}$
oAM26	telomere PCR primer for 7L	CGG ATC CCA GAG TAG AGG TAG
oBL258	-6bp primer-forward	GTG TGT AGT GAT CCG AAC TCA
oBL259	-6bp primer-reverse	GCA TAT TGA TAT GGC GTA CGC ACA CGT
oHP43	-500bp primer-forward	TAA GGA TTC GAA CGT GAT CC
oHP45	-500bp primer-reverse	ACA TAA GCG TAT CCA ATT TTG ACA
oHP27	-1000bp primer-forward	ATT AAA GAC ACC GCC AAG CTT
oHP46	-1000bp primer-reverse	AGG AAT GAT CTT GGA AAT CGA TC
oHP31	-2000bp primer-forward	GAC CCG GAA TTA CAA TAC AAT GC
oHP48	-2000bp primer-reverse	TTC GGT GTG CGT TTG TGT
oHP35	actin primer-forward	GAT TTG GCC GGT AGA GAT TTG A
oHP50	actin primer-reverse	TTC CTT GAT GTC ACG GAC AA
oHP6	UAS forward	CAT GAA GCT TAA GCC GCC GAG CGG GTG ACA G
oHP7	UAS reverse	CAT GAA GCT TAG GAA CGC GAC CGG TGA AGA C
oBL207	Telomere probe	CAC CAC ACC CAC ACA CCA CAC CCA CA
oBL358	Telomere PCR primer for 1L	GCG GTA CCA GGG TTA GAT TAG GGC TG
oBL360	Telomere PCR primer for 6R	AAA TGA GGA CTG GGT CAT GG
oBL361	Telomere PCR primer for Y	TTA GGG CTA TGT AGA AGT GCT G
oBL17	URA3 forward primer	GAA GAT CTA TGT CGA AAG CTA CAT ATA AGG
oBL18	URA3 reverse primer	AGC TTT GTT TAA ACT TAG TTT TGC TGG CCG CAT CTT C
oBL292	Actin forward primer	CCC AGG TAT TGC CGA AAG AAT GC
